# The Effect of a Vegan Diet on the Coverage of the Recommended Dietary Allowance (RDA) for Iodine among People from Poland

**DOI:** 10.3390/nu15051163

**Published:** 2023-02-25

**Authors:** Agata Zaremba, Anna Gramza-Michalowska, Kunal Pal, Krystyna Szymandera-Buszka

**Affiliations:** 1 Department of Gastronomy Science and Functional Foods, Faculty of Food Science and Nutrition, Poznań University of Life Sciences, Wojska Polskiego 31, 60-624 Poznań, Poland; 2 Department of Biotechnology and Medical Engineering, National Institute of Technology, Rourkela 769008, India

**Keywords:** iodine sources, iodized salt, vegan, omnivores, iodine deficiencies, food consumption

## Abstract

The aim of this research was to estimate the effect of a vegan diet on the Recommended Dietary Allowance (RDA) coverage for iodine in people from Poland. It was hypothesized that the problem of iodine deficiency is a concern, especially among vegans. The survey study was conducted in the years 2021–2022 on 2200 people aged 18–80 with omnivore and vegan diets. The exclusion criteria in the study were pregnancy and lactation. The study found that the coverage of RDA for iodine among people with a vegan diet was lower than among people with an omnivore diet (*p* < 0.05); 90% of the participants with a vegan diet had an iodine intake below 150 µg/day. Plant-based dairy and meat analogs were consumed by vegans frequently and in large portions, but none were fortified with iodine. It was found that iodized salt was each group’s primary source of iodine. However, it was observed that the iodine supply from this source was limited among vegans, especially in female subjects, who consumed less salt and smaller portions of meals. That is why consideration should be given to the iodine fortification of plant-based foods commonly consumed by vegans.

## 1. Introduction

In recent years, there has been an increase in interest in plant-based diets. Earlier consumer studies showed that from 2019 to 2020, as many as 5 million consumers in the United States shifted to avoiding meat altogether, becoming either vegetarians or vegans [1].

The popularity of vegan cuisine is most evident in the UK, Australia, Israel, Austria, New Zealand, and Germany. The Swedes, the Swiss, and Canadians are also more likely to follow a plant-based diet [2]. According to the Vegan Society, 2021, 6% of the US population follows a strict vegan diet, compared with up to 4% in Europe and 13% in Asia [3]. In Israel, 5% of the population declares themselves vegans [4]. In Poland, the sale of meat products fell by 7.5% over the last three years, whereas the sale of plant-based foods grew by 30% for dairy products and 60% for meat products [5]. Among the reasons for switching to a plant-based diet are environmental protection, altruism towards animals, or the desire to improve wellbeing [6].

Veganism is considered the most stringent form of vegetarianism [7]. Due to sometimes very significant differences in the selection of products, the nutritional status of individual nutrients in people who adhere to plant diets differs from that in people who consume meat and other animal products [8].

The health benefits of using plant-based diets include reducing the risk of sclerosis, metabolic syndrome, or certain cancers [8,9]. At the same time, despite the many positive aspects of limiting animal products in the diet, risk factors should also be considered. Numerous studies show that a plant-based diet, especially vegan, is characterized by an insufficient supply of ingredients such as protein, ω-3 fatty acids, vitamin B12, iron, vitamin D, and calcium [9,10,11]. Less numerous studies also indicate deficiencies in zinc and iodine [12,13].

Iodine is necessary for the proper development and functioning of the human body due to its participation in the synthesis of thyroid hormones (T3, T4). Moreover, it affects basic metabolism [14,15,16]. A deficiency of an element that affects so many processes in the body leads to a spectrum of symptoms classified as iodine deficiency disorders (IDD), including goiter and hypothyroidism. Chronic, severe iodine deficiency in children causes cretinism or developmental delay [17,18]. The intake of stable iodine is also considered an effective countermeasure for reducing the risk of thyroid cancer in the case of a possible release of radioactive iodine following a nuclear accident [19]. The recommended daily iodine intake for adults was established at 150 µg [20]. Studies suggest that iodine content in vegetarian diets may be inadequate, but adherence to a balanced vegetarian diet need not lead to iodine deficiency. The iodine intake by the vegan Japanese population is among the highest in the world [21]. However, younger Japanese vegans tend to consume food with low iodine content [22,23]. The latest statistics indicate that 56.9% of Europeans have an insufficient intake of iodine [20,24,25]. The diet of Poles can also be generally low in iodine [17]. This is because primary sources of iodine are excluded from the diet. The primary naturally occurring sources of iodine in the diet are animal products such as fish, seafood, eggs, milk, and dairy products [26]. In contrast, the content of iodine in plant products is strongly correlated with the content of this element in the soil [25]. The primary plant source of iodine may be algae, as the content of this element is high in them [25]. Earlier research confirms that the highest iodine intake was recorded for females following vegan diets with regular consumption of seaweed [27,28]. However, seaweed is not customarily consumed in the Western diet, although its popularity is increasing. As there is also no culture of consuming these types of products in Poland, they are not present in traditional gastronomy. In addition, the possibilities of enriching food with algae are limited due to legal restrictions [29]. Plant-based dairy and meat analogs are increasingly popular, but they are naturally low in iodine, so they cannot be considered nutritionally equivalent to milk.

To minimize the risk of deficiency of this element caused by insufficient amounts in the diet, programs to fortify food with iodine are carried out in many countries around the world. One of the most common fortification strategies, also used in Poland, is salt iodization. The salt’s iodine fortification consists of soaking the product in a solution of potassium iodide or potassium iodate at an appropriate concentration. In Poland, these concentrations are legally regulated and amount to (30 ± 10) mg/kg of potassium iodide (KI) or (39 ± 13) mg/kg of potassium iodate (KIO_3_). The program of obligatory salt iodization in Poland has had very good results. In 2002, Poland was classified by the WHO as a country with sufficient iodine supply at the population level [20,30]. The analysis of iodine consumption shows that iodized salt was the primary source of iodine [26]. However, in 2006, the World Health Organisation introduced a recommendation to limit salt intake to 5 g/day, as it is a risk factor for atherosclerosis and hypertension. Consequently, the iodine supply from this source can be limited. Therefore, appropriate activities must be undertaken to promote the joint implementation of programs for reducing sodium intake and eliminating IDD through salt iodization [31].

Considering the above, this research aimed to estimate the effect of a vegan diet on the coverage of the RDA for iodine and evaluate the prevalence of the risk of inadequate iodine intake (below 41% of RDA for iodine) among people aged 18–80 from Poland. It was hypothesized that the problem of iodine deficiency in the diet especially concerns the vegan group. Our study also assessed the impact of variables concerning age, sex, and education level. We estimated the relative contributions of iodized salt and iodine introduced by foods to the total iodine intake.

## 2. Materials and Methods

### 2.1. The Survey Questionnaire

The study was conducted using an anonymous questionnaire based on the KomPAN questionnaire [32] and the author’s questions. The author’s questions were validated. The average correlation coefficients for the intake frequency of individual products were r = 0.81, and for product quantities, the coefficients were r = 0.79.

The survey questionnaire was completed from January 2021 to June 2022. A total of 2341 questionnaires were sent in and registered in the system; of these, 2200 questionnaires were correct (94%).

The questionnaire was made available in electronic form via Google Forms. The surveys were distributed on Polish internet forums for vegans, nutrition, and nutrition for the elderly. Questionnaires were also completed in outpatient family doctor clinics using the investigator’s computer.

No ethical approval was required for this study. Participants were informed about the study’s aim and that their participation was entirely voluntary; therefore, they could stop the analysis at any point, and the responses were anonymous. The authors did not ask for sensitive data and personal information. Formal dependence was not used in recruiting subjects for the study.

The questionnaire contained questions about the type of diet, the frequency and amount of consumed products, and the types of culinary processes used when preparing products with iodized salt (allowing for a complete determination of the iodine content in the diet from salt) (Table S1 and S2). The information on the method of food preparation (the types of culinary processes used) consisted of how salt was added to the food (or whether salt was added at all) at the end of heating, halfway through heating, and at the beginning of water heating. Based on the authors’ preliminary research (unpublished data) on the content of iodine in iodized salt cooked under varying conditions, iodine losses were assumed. Adding salt at the end of heating (losses of 0%), adding salt halfway through heating (losses of 25%), and adding salt at the beginning (losses of 50%) of water heating. The conversion factor assumed the cooking of meats (for approximately 30 min), potatoes (for approximately 20 min), pasta, rice, and groats (for approximately 10 min), and losses were taken into account only for these culinary processes. For the purpose of calculations of iodine from salt, only salt as a household additive was used. Food products for which the frequency and amount of consumption were asked in the questionnaire were: eggs; dairy products (butter, milk, yogurt, kefir, buttermilk, sour milk, sour cream, cottage cheese, cheese, e.g., Emmental, Mozzarella, Cheddar, Gouda, Brie, Gorgonzola); vegetable drinks; vegan products and dishes (vegan burgers, vegan sausages, bacon, and meat free ham); soy and its products (tofu, tempeh); legume seeds (peas, beans, chickpeas, lentils); vegetables (cabbage, Brussels sprouts, leek, garlic, onion, pepper, tomato, asparagus, and spinach); fruits (banana, apple, blueberries, strawberries, raspberries, peaches, apricots, tangerines, figs, plums); algae; poultry (chicken, turkey, duck, goose); red meat (pork, beef); cold cuts; fish (salmon, mackerel, tuna, cod, haddock, plaice, pollock, sprat, herring, trout); sushi; bread (light, wholemeal, graham, rye, wheat bread); and grain products (buckwheat, millet, barley, white rice, bran, and flakes).

Based on the frequency and amount of consumption of particular products as declared by the respondents, as well as on the iodine content in the products, the weekly iodine consumption and the percentage coverage of the demand for this element were estimated. Iodine content in the products was determined based on the tables of composition and nutritional value [33,34]. For iodine calculations from salt, only salt as a household additive was used. For iodine calculations from plant-based dairy and meat analogs—none of them were fortified with iodine, and no iodine content was assumed. Taking into account the average iodine content in 100 g of the product, the content of this element was converted into the usual portion size provided by the respondents. Then, the content of iodine was converted into weekly consumption based on the frequency of consumption, according to an 8-point scale declared by the respondents. The following conversion scheme was adopted: 1—‘less often/never’ means consumption of 0 servings per week; 2—‘several times a year’ means consumption of 0.1 servings per week; 3—‘once a month’ means consumption of 0.25 servings per week; 4—‘several times a month’ means consumption of 0.75 servings per week; 5—‘once a week’ means consumption of 1 serving per week; 6—‘several times a week’ means consumption of 3 servings per week; 7—‘once a day’ means consumption of 7 servings per week; 8—‘several times a day’ means consumption of 21 servings per week. When calculating the coverage of iodine demand, the daily dose of this element was assumed to be 150 µg, i.e., 1050 µg per week.

### 2.2. The Group of Respondents

The group selection was random, with stratified sampling taking into account the sampling of Polish society in terms of the type of diet, sex, and age. The exclusion criteria in the study were age (under 18) and pregnancy and lactation. The study population consisted of 2200 people of both sexes (i.e., 49% males and 51% females) aged 18–80 years in different areas of Poland. The subjects were on an omnivore diet and a vegan diet (Table 1).

Regarding education, the largest group was people with higher education (41.6%); people with vocational education accounted for 30.36% of the study group, and people with secondary education 28.05%. The subjects were divided into age groups: 18–30; 31–40; 41–50; 51–60; 61–70; and 71–80 (Table 1).

### 2.3. Statistical Analysis

The data were analyzed with Statistica (Software v. 13, StatSoft, Tulsa, OK, USA). The calculations were performed at a confidence level of 0.95, and the maximum error rate was set at 0.05. One-way analysis of variance (ANOVA) was carried out to compare the means of the groups (covering RDA for iodine) at a significance level of *p* < 0.05. Then, post hoc Tukey’s test was applied. Interdependencies between the qualitative variables (diet, age, sex, education level) were determined with the chi-squared independence test at α = 0.05, which showed differences between the diet and sex.

The logistic regression analysis (the odds ratio—OR) was applied to predict the covering of the RDA for iodine among people with a vegan diet. The ranges assumed were: high (above 100% RDA); moderately high (81–100% RDA); medium (60–80% RDA); low (41–60% RDA); very low (21–40% RDA); or alarmingly low (below 20% RDA) RDA for iodine depending on the people with a vegan diet and among women.

## 3. Results

### 3.1. Iodine Intake

The recommended amount of iodine that adults should consume each day is 150 µg.

Unfortunately, approximately 50% of respondents from Poland declared the consumption of food products that allowed for the coverage of RDA for iodine below 100%.

**Figure 1:**
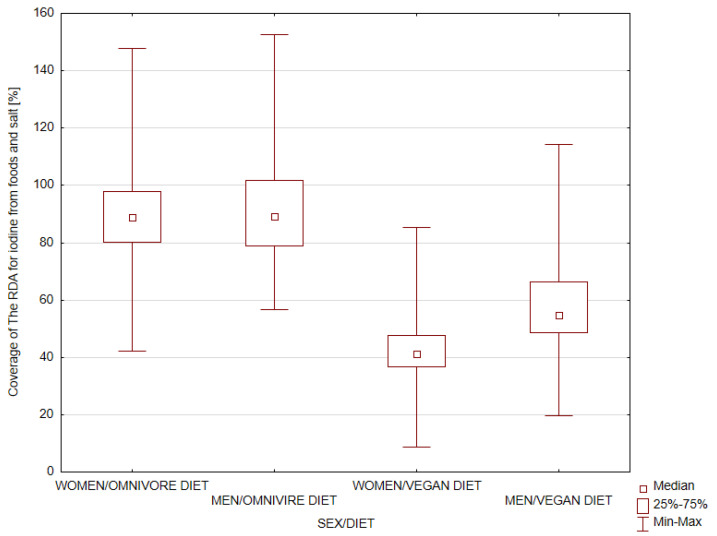
Contribution percentage of food sources to the coverage of total RDA for iodine among women and men in different diet groups (vegan, omnivore) aged 18–80 from Poland.

The research results showed that the intake estimate of iodine from foods in the Polish population ranged from 9 to 160 µg/day, i.e., 67–1120 µg/weekly, which is 8 to 152% of RDA for iodine intake (Figure 1).

The covariance analysis (Table 2) showed a statistically significant influence (*p* < 0.05) of the predictors, i.e., the type of diet, sex, and age, on the amount of coverage of the RDA for iodine.

Taking the strength of statistically significant influence (Table 2) into account, the predictors can be ranked from the most to the least impact in the following order: the type of diet > sex > age > education level. The statistical analysis showed that the diet’s most significant effect was confirmed.

A chi-squared independence test (Table 3) also confirmed the strongest relationship between the type of diet (*p* < 0.05) and the coverage of RDA for iodine. People with a vegan diet had a significantly lower intake of iodine from food than people with an omnivore diet (*p* < 0.05; Table 3).

**Figure 2:**
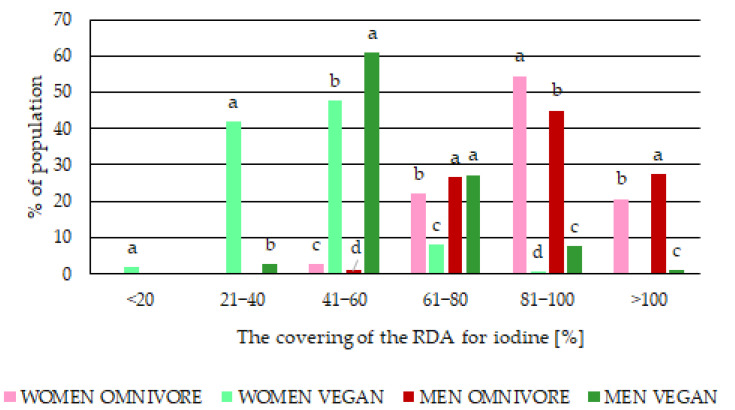
Contribution percentage of the coverage of total RDA for iodine according to ranges (above 100% RDA; 81–100% RDA; 60–80% RDA; 41–60% RDA; 21–40% RDA; below 20% RDA) among women and men in different diet groups (vegan, omnivore) aged 18–80 from Poland; different letters (a–d) denote a significant difference at *p* < 0.05 (one-way ANOVA, and post hoc Tukey test) between men and women in different diet groups (vegan, omnivore) within a given RDA ranges.

In vegan people (Figure 1), the estimated iodine intakes ranged from 8 µg/day to 114 µg/day, but on average, 51% RDA for iodine (Median value = 48 µg/day). In people with an omnivore diet, the intake was 90% RDA for iodine on average (Median value = 79 µg/day). In the group of participants with a vegan diet, 90% had an iodine intake below 150 µg/day. Only 1% of vegans had an intake of iodine above 150 µg/day (Figure 2). For omnivore participants, the percentage with an iodine intake below 150 µg/day was 76%. The estimated iodine intake above 150 µg/day was 24% of omnivores.

To maintain homeostasis and hormone synthesis, the thyroid absorbs 60 µg/day of iodine when the supply is sufficient [35]. Therefore, logistic regression analysis was applied to predict very low (21–40%) or alarmingly low (below 20%) coverage of the RDA for iodine among people with a vegan diet (Table 4; Figure 2). The logistic regression analysis indicated a strong relationship between alarmingly low (below 20%) and very low (21–40%) coverage of RDA for iodine and a vegan diet (Table 4).

A chi-squared independence test also showed a relationship between the sex of the respondents and the covering RDA for iodine, especially in the vegan group (Table 3).

It was found that 64% of men vegan participants had an iodine intake below 61% of RDA for iodine, and 94% of vegan women had an iodine intake below 61% of RDA iodine, including 43% who had below 41% RDA iodine.

### 3.2. Iodine Sources

**Figure 3:**
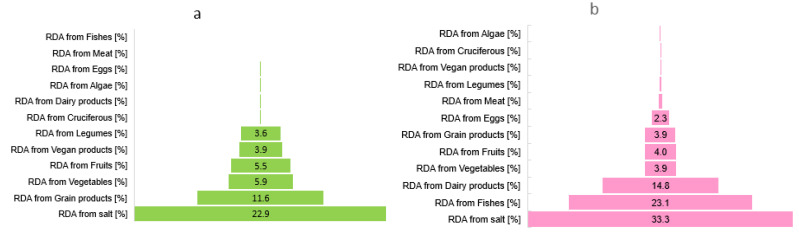
Ranked covering of the RDA for iodine from products among women and men with the vegan diet (**a**) and the omnivore diet (**b**) aged 18–80 from Poland.

**Figure 4:**
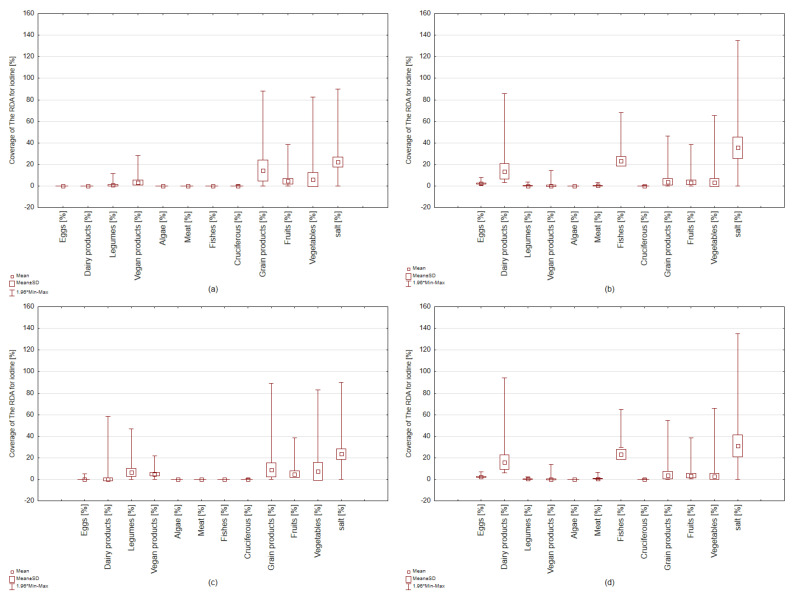
Contribution percentage of RDA for iodine from product groups among women with the vegan diet (**a**) and the omnivore diet (**b**) and men with the vegan diet (**c**) and the omnivore diet (**d**) aged 18–80 from Poland.

Figure 3 and Figure 4 present the sources of iodine in people with a vegan diet and the omnivore diet (men and women). It was found that estimated iodine intake from food products (without iodine salt) contributed 31% among women and men with vegan diets (Figure 3a). In women and men with an omnivore diet, this intake contributed 53% of RDA coverage for iodine (Figure 3b).

It was found that dairy products were only consumed by the people surveyed with an omnivore diet, both in women and men (Figure 3b and Figure 4b,d). These products met 17% of the iodine-recommended daily allowance among women and 19% among men. Sea fish and seafood (rich iodine sources) contributed to the iodine intake of only 23%, but only among people with an omnivore diet. A low frequency of consumption of these products (twice a month on average) was found in both women and men. In women with an omnivore diet, egg products contributed 2% of total iodine intake and meat products 0.5% (Figure 4b,c), while in men, 4% and 0.6% of intake, respectively. Fruit and vegetables contributed approximately 8% of the total iodine intake in both women and men with an omnivore diet.

Our studies showed a high prevalence of iodine deficiency among vegans, especially in women of all ages (Figure 4a,c). Iodine content in food of plant origin is lower than in animal origin due to the low iodine concentration in soil.

Vegan dairy products were frequently consumed by the surveyed women with a vegan diet but could satisfy only 4% of the RDA for iodine. These products were not iodine-fortified. A total of 40% of women and 10% of men consumed them daily, an average of 200 g/day. Similarly, vegan meat substitutes (i.e., vege-ham, vege-sausage, vege-burger) were frequently consumed by the surveyed vegan population, especially men. Unfortunately, with these products, women could meet a mere 2% of the recommended daily allowance and men 3%. These products were not iodine-fortified. Legumes and their fermented products contributed 2% of total iodine intake among vegan women and 3% among men. These products were frequently and in large portions consumed by the surveyed women and men but were not iodine-fortified. Seaweed (rich iodine sources) contributed to an iodine intake of only 1%. A low frequency of consumption of these products (once a month on average) was found.

Fruit and vegetables contributed approximately 11% of the total iodine intake in both women and men with vegan diets. High consumption of cruciferous vegetables, especially broccoli, cabbage, and cauliflower, was found. Unfortunately, a high frequency of consumption of these products was found (daily), especially among women with a vegan diet. This can decrease iodine absorption and also contribute to the incidence of thyroid cancer [36]. In vegan women, cereal products (groats, pasta, bread) contributed 8% of total iodine intake, and among men, 9% (Figure 4). Whereas in both women and men with an omnivore diet, cereal products (groats, pasta, bread) intake contributed 5% of total iodine intake. The iodine intake from these products contributed to the covering of RDA for iodine 14% and 8% among women and men with vegan diets, respectively, and 4% with an omnivore diet.

It was found that iodized salt was each group’s primary source of iodine. However, the iodine supply from this source was found to be limited among vegans, especially concerning women. Women (vegan and omnivore) declared average salt consumption at the level of 3 g/day. Among men, salt consumption was similar to women, 3 g/day.

**Figure 5:**
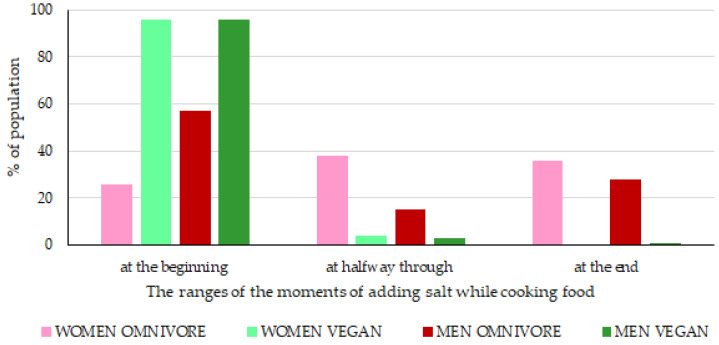
The time (at the beginning, halfway through, the end) to add salt to food while cooking among women and men with the vegan diet and the omnivore diet aged 18–80 from Poland.

However, it is known that a significant amount of iodine (10–40%) in salt may be lost during culinary processing [37,38,39,40,41,42]. Therefore, our research (Figure 5) was also concerned with analyzing habits related to salt being added to the food (at the end of heating, halfway through heating, at the beginning of water heating, or no salt was added). Our study found that 96% of vegan women and 26% with an omnivore diet declared adding iodized salt at the beginning of water heating, e.g., to potatoes or pasta. It is not a good practice because the loss of iodine can be as high as 20–50%. Therefore, for those who indicated adding salt at the beginning of cooking, the iodine content was calculated at 50% of the initial content. Unfortunately, only 36% of women with an omnivore diet declared adding salt at the end of heating. None of the women with a vegan diet declared adding salt at the end of heating. Hence, the differences in RDA for iodine from salt. Among the vegan women group, it was found that 22% of RDA for iodine was from iodized salt, and among women with an omnivore diet, 36% (Figure 4). Similarly, it was found that 96% of vegan men and 57% of omnivore men also declared adding iodized salt at the beginning of water heating.

Therefore, our studies showed that in the Polish population average daily consumption of iodized salt met 28% of the recommended allowance of iodine. It was found that those who did not consume iodized salt (*n* =10) ingested an estimated 14% of the RDA for iodine.

## 4. Discussion

The present study found that people with a vegan diet had a significantly lower intake of iodine from food than people with an omnivore diet. Low iodine intakes have also been reported in other European populations, especially vegans [43,44,45,46]. Iodine nutrition is also a growing issue within industrialized countries, including the US, due to declining iodine intake, partly attributable to changing dietary patterns and food manufacturing practices [26]. A recent national iodine survey found Israel’s population to be mildly iodine deficient, possibly due to unmonitored changes in the food content of dietary iodine [47].

In our research, we confirmed that vegan women and men from Poland have low iodine intake, regardless of age and education level.

Krajcovicová–Kudlacková et al. (2003) and Groufh–Jacobsen (2020) confirmed similar tendencies in their research. They found that vegetarian groups from Europe, mainly vegan, tended to have the lowest iodine intake [48,49].

In our research, we confirmed that women, especially those with a vegan diet, have worse compliance with the iodine recommendations than those with an omnivore diet. The trend of lower iodine intake in women than men, especially vegans, was confirmed by Groufh–Jacobsen (2020) and Hatch–McChesney (2022) [26,48]. The low intake of iodine in women is in agreement with earlier studies showing a low iodine intake among women in Norway [44,50,51,52,53,54,55]. According to Eveleigh et al. (2020), iodine intakes for moderate vegans (vegetarians), vegetarians, and pescatarians from some populations were similar between sexes but also were below RDA for iodine [43].

Our studies confirmed that in people with an omnivore diet, the most important iodine sources were dairy products, especially yogurt, cheeses, and fish, especially sea fish. Similar trends were confirmed among Norwegians by Carlsen (2018) [56,57,58,59]. In the UK, the main sources of dietary iodine are cow’s milk and dairy products (2022). Similarly, Israel’s milk and dairy products are iodine rich [42]. Brzóska et al. (2015), after analyzing the iodine content in milk in various regions of Poland, found that the average iodine content in drinking milk in Poland, taking into account its losses as a result of pasteurization, is at the level of about 100–200 µg/l. The authors confirm that Polish milk may be an important element of IDD prevention [60]. Thanks to the iodine content in milk, its products (cheese, fermented milk drinks) are also a source of this element. These have a higher iodine content due to the higher content of dry matter [61,62]. On average, cheese contains 37.5 mcg of iodine per 100 g of cheese; 100 g of Cheddar cheese provides 32% of the daily requirement for iodine.

Our research confirmed an earlier study that while the intake of white cheese has increased, there has been a decrease in the intake of milk [62]. Our research also drew attention to the tendencies of lower frequency and amount of milk consumed among young people. These trends and even stronger ones were confirmed in earlier studies [63,64,65,66,67,68]. However, an increasing frequency of consumption of those gaining in popularity, such as plant-based alternatives, was observed.

Our study showed that people on a vegan diet consumed significant amounts of milk-alternative drinks. Dinewa et al. (2021), analyzing the consumption of cow’s milk and milk-alternative drinks in the UK population, found that cow milk consumers have higher UIC medians than those consuming milk-alternative drinks. On the basis of the collected data, it was proved that people who consumed milk-alternative drinks were at risk of deficiency of this element [26,69,70]. These authors also point to the need to fortify milk-alternative drinks so that they provide at least as much iodine as cow’s milk [71].

Due to their high consumption, chicken eggs can be a source of iodine in the diet [72]. This was confirmed by our research among omnivore subjects. On the basis of our data, it was proved that people who consumed eggs (a few times a week) were less at risk of deficiency of this element. However, the authors point to the seasonal variability of iodine content in eggs due to feed.

Sea fish are a rich source of iodine [34]. Lower iodine intake was found among women compared to men, which can also be seen in an earlier study [73,74]. These studies showed that the consumption of fish products was so low that it only covered 10% of the iodine requirement [73].

Vegans consume plant-based foods and omit all types of animal products.

Algae are a plant product that provides significant amounts of iodine. Earlier research confirms that the highest iodine intake was recorded for females following a vegan diet with regular (daily) consumption of seaweed [75,76]. It has recently become popular in UK food products as a whole and as a functional ingredient [27]. However, in our study, a low frequency of consumption of these products (once a month on average) was found. Moreover, iodine content significantly differs between seaweed species consumed [28].

Unfortunately, in the study group, vegans were found to have a high consumption of plant-based food groups (fruit, vegetables, legumes, tubers, cereals, and grains) [55,77,78,79], along with tofu and soya-based products that naturally have a low iodine content. The Polish market also does not offer biofortified vegetables.

In our research, a high frequency of consumption of cereal products was found (several times a day), especially among men. In vegan women, cereal products (groats, pasta, bread) contributed 20% of total iodine intake, and among men, 22%. However, these were not as high iodine sources as in the research among Norwegians [55]. The Norwegian and American markets offer a wide range of iodized bread with iodized salt [34,55]. In Switzerland, iodine-fortified bread is a significant contributor to dietary iodine and can be consumed by all dietary groups [80]. Unfortunately, bread does not introduce high iodine amounts into the diet though it is a staple food in Poland’s population.

Our research confirms an earlier study [46] which indicated that the population could not reach the recommended iodine intake from food sources [37,55,62,81]. The most practical and cost-effective way to provide iodine supplementation to deficient populations is with iodized salt, as advocated by several international organizations such as the WHO, United Nations Children’s Fund, and International Council for Control of Iodine Deficiency Disorders [82,83].

However, it was found that the iodine supply from this source is limited, especially concerning vegan women. This follows a reduction in iodized salt consumption, possibly influenced by efforts to reduce salt consumption in the general public [84,85]. Additionally, bad habits exist related to the use of salt in the household.

Our study found that over 80% of the respondents declared adding iodized salt at the beginning of water heating, e.g., to potatoes or pasta. Therefore, in our research, the amount of salt (for declaring adding iodized salt at the beginning of water heating) added during the processing was reduced to 50%.

A strength of this study was the sample size of over 2202 participants (1000 vegan, aged 18–80), compared to previously conducted studies on vegans and vegetarians [51,86,87]. Another strength is the assessment of iodine intake with methods including the assessment of iodine intake from iodized salt. The earlier study did not identify adjusting iodine intakes to account for the types of culinary processes used.

The main limitations were that the presented study did not take into account the place of residence of the respondents and regional differences in the frequency of consumption of products [88], and the lack of measurements of urinary iodine concentration. Therefore, further research should focus on confirming the obtained dependencies using such measurements.

## 5. Conclusions

The study found that the coverage of the Recommended Dietary Allowance (RDA) for iodine among people with a vegan diet was lower than among people with an omnivore diet. A total of 90% of the participants with a vegan diet had an iodine intake below 150 µg/day. The results also showed a relationship between the vegan diet of women and the lowest coverage of the RDA for iodine, below 41%. Plant-based dairy and meat analogs were consumed frequently and in large portions by vegans, but none were fortified with iodine. It was found that iodized salt was each group’s primary source of iodine. However, the iodine supply from this source was also limited among vegans, especially among women. This follows a reduction in iodized salt consumption, unfavorable habits related to the use of salt in the household, and smaller portions of meals. That is why consideration should be given to iodine fortification of foods commonly consumed by vegans, including plant-based milk alternatives, within the acceptable dosage range.

## Figures and Tables

**Table 1:** Distribution [%] of respondents by age groups (*n* = 2200).

Diet	Sex	Educational Level	Age	Distribution	Diet	Sex	Educational Level	Age	Distribution
Omnivorous	Women *n* = 541 *p* < 0.05	Vocational *n* = 151	18–30	21	Vegan	Women *n* = 564 *p* < 0.05	Vocational *n* = 160	18–30	20
			31–40	25				31–40	25
			41–50	27				41–50	27
			51–60	23				51–60	29
			61–70	24				61–70	27
			71–80	31				71–80	32
		Secondary *n*= 150	18–30	25			Secondary *n* = 171	18–30	27
			31–40	25				31–40	26
			41–50	24				41–50	28
			51–60	20				51–60	23
			61–70	31				61–70	40
			71–80	25				71–80	27
		Higher *n* = 240	18–30	31			Higher *n* = 233	18–30	32
			31–40	29				31–40	29
			41–50	34				41–50	33
			51–60	42				51–60	40
			61–70	50				61–70	49
			71–80	54				71–80	50
	Men *n* = 531 *p* < 0.05	Vocational *n* = 175	18–30	34		Men *n* = 564 *p* < 0.05	Vocational *n* = 182	18–30	33
			31–40	29				31–40	32
			41–50	12				41–50	11
			51–60	52				51–60	35
			61–70	13				61–70	31
			71–80	35				71–80	40
		Secondary *n* = 135	18–30	18			Secondary *n* = 161	18–30	20
			31–40	27				31–40	31
			41–50	9				41–50	16
			51–60	35				51–60	28
			61–70	14				61–70	30
			71–80	32				71–80	36
		Higher *n* = 221	18–30	29			Higher *n* = 221	18–30	23
			31–40	35				31–40	38
			41–50	28				41–50	23
			51–60	61				51–60	35
			61–70	24				61–70	52
			71–80	44				71–80	50

**Table 2:** Statistical significance of predictors of covariance models for changes in the average weekly coverage of demand for iodine.

Predictors	SS	*df*	MSE	F-Value	*p*-Value	Post Hoc Power α = 0.05
Diet	8.448 × 10^5^	1	8.448 × 10^5^	3.550 × 10^3^	<0.05	1.000
Sex	3.832 × 10^4^	1	3.832 × 10^4^	6.335 × 10^1^	<0.05	1.000
Age	2.635 × 10^4^	5	5.269 × 10^3^	8.618 × 10^0^	<0.05	0.999
Education	3.457 × 10^3^	2	1.729 × 10^3^	2.783 × 10^0^	0.063	0.549

SS—Statistical Significance; *df*—degrees of freedom; MSE—mean sum of squares.

**Table 3:** Dependence between the covering the RDA for iodine and selected factors (diet; education, sex, age) among people aged 18–80 (chi-square independence test).

Type of Group	Test Value	The Degrees of Freedom (*df*)	*p*-Value
Diet	1.478 × 10^3^	5	<0.05
Sex	2.267 × 10^1^	5	<0.05
Age	1.164 × 10^1^	25	<0.05
Education	2.451 × 10^−2^	10	0.094

**Table 4:** Results of logistic regression analysis to the covering the Recommended Dietary Allowance of iodine among people with vegan Diet.

Level	Odd Ratio (OR)	95% CI
TOTAL GROUP		
<20	9.848 × 10^5^	13.801–31.622
21–40	2.887 × 10^7^	0.444–17.171
41–60	6.597 × 10^1^	0.348–4.189
61–80	6.656 × 10^−1^	−0.407–0.642
81–100	4.283 × 10^−2^	−3.150–1.991
>100	1.970 × 10^−2^	0.000–0.015
WOMEN		
<20	1.805 × 10^6^	14.412–31.623
21–40	7.195 × 10^7^	0.444–11.809
41–60	3.457 × 10^1^	0.348–3.543
61–80	3.042 × 10^−1^	−1.190–0.642
81–100	4.459 × 10^−3^	−5.413–1.991
>100	0.000 × 10^0^	−17.082–1.471
MEN		
<20	1.776 × 10^5^	12.091–31.623
21–40	2.920 × 10^6^	0.444–14.887
41–60	1.633 × 10^2^	0.348–5.095
61–80	1.039 × 10^0^	0.038–0.642
81–100	0.101 × 10^−1^	−2.294–1.990
>100	3.314 × 10^−2^	−3.407–1.471

## Data Availability

Not applicable.

## Supplementary Materials

The following supporting information can be downloaded at: https://www.mdpi.com/article/10.3390/nu15051163/s1. Table S1: Questions about the consumption of products as iodine carriers and types of culinary processing for meats product; Table S2: Questions about how salt was added to the food (time of cooking).
