# Supplementary material for: The Effect of a Vegan Diet on the Coverage of the Recommended Dietary Allowance (RDA) for Iodine among People from Poland
Source: Nutrients. 2023 Feb 25;15(5):1163. doi: 10.3390/nu15051163 (PMC10005417; doi:10.3390/nu15051163)
Supplement: Supplementary file 1 [file nutrients-15-01163-s001.zip › nutrients-2226127-supplementary.pdf]

Supplementary Tables S1. Questions about the consumption of products as iodine carriers and types of culinary processing for meats product.

Put the answers down into the table: how often you consume the product in the table and enter the amount of the most frequent consumption [g or ml]

	several times a day	once a day	several times a week	once a week	several times a month	once a month	'several times a year	less often / never
**Dairy produkt**								
butter								
milk								
yoghurt/kefir/buttermilk drinks								
sour milk								
yoghurt/kefir/thick fermented (e.g. Greek yoghurt)								
sour cream								
cottage cheese								
cheese e.g. Cottage, Mozzarella, Feta								
cheese e.g. Brie, Camembert, Livarot								
cheese e.g. Emmental, Gouda, Edam, Cheddar								
cheese e.g. Grana Panado, Parmesan								
other cheeses, name...								
other dairy product, name...								
other dairy product, name...								
Eggs								
**Dairy alternative -** please specify the type of milk (e.g. rice) and the company								
coconut drink								
rice drink								
oat drink								
soy drink								
almond drink								
other plant-based drinks, name and the company ……								
plants CREAM FOR COFFEE name and the company ……								
Dairy alternative cheeses name and the company ……								
plants CREAM FOR COFFEE INSTANT name and the company ………								
other dairy alternative, name and the company ……								
Vegan products and dishes - please specify the name of product and the company								
vegan burgers, name and the company ……………….								
vegan sausages, name and the company ……………….								
bacon , name and the company ……………….								

meat-free ham, name and the company ……………….								
products type tofu, name and the company ……………….								
other products type tempeh, name and the company ……………….								
other products in this group name and the company ……………….								
Legume seeds								
peas								
beans								
chickpeas								
lentils								
other legume seeds, name…………………..								
soybean paste g.e. SSAMJANG PASTE. name and company …..								
legume seeds paste name and company …..								
legume seeds bars, name and company's …..								
soy sauce and other, name and company…….								
Nuts								
hazelnuts								
walnuts								
peanuts								
cashew nuts								
pistachios								
Other nuts, name ….								
cream with nuts e.g. peanuts, name								
peanut bars, name and company ……..								
Onther products with nuts, name and company …								
Grain products								
Crispbread, name and company …								
toasted bread, name and company …								
light bread name and company ….								
wheat bread								
wheat-rye bread								
graham bread								
rye bread								
Onther bread, name …								
barley groats (whole grains)								
barley groats (broken grains, e.g. Masurian groats)								
porridge								
buckwheat groats								
millet groats								

cous cous								
bulgur groats								
other groats, type……								
long-grain white rice								
rice, e.g. Basmati								
brown rice								
wild rice								
other rice, name ….								
pasta, name								
whole-grain pasta, name…								
Other special pasta (e.g. with vegetables, name ……..								
Bran, name and company ….								
flakes wholemeal , name and company …								
other flakes , name and company ….								
cereal bars, name and company …….								
Other grain product, name and company								
**Vegetables**								
cauliflower								
broccoli								
cabbage								
brussels sprouts								
beetroot								
tomatoes								
raw cucumber								
pickled cucumber								
potatoes								
pumpkin								
zucchini								
pepper								
leek								
garlic								
onion								
pepper								
asparagus								
spinach								
lettuce								
lamb's lettuce								
parsley root								
celery root								
other vegetables, name …								
**Fruits**								
banana								
apple								
pear								
blueberries								
strawberries								
raspberries								

peaches								
apricots								
tangerines								
orange								
figs								
grape								
plum								
kiwi								
tangerine								
pineapple								
red currant								
black currant								
mango								
Other fruits, name …								
**Algae and products with the addition of algae**								
WAKANE leaves, name and company…								
WAKANE drought, name and company….								
WAKANE tablets, name and company…								
WAKANE drinks, name and company….								
NORI leaves, name and company…								
NORI drought, name and company….								
NORI tablets, name and company…								
NORI drinks, name and company….								
KOMBU leaves, name and company…								
KOMBU drought, name and company….								
KOMBU tablets, name and company…								
KOMBU drinks, name and company….								
OTHER leaves, name and company…								
OTHER drought, name and company….								
OTHER tablets, name and company…								
OTHER drinks, name and company….								
salads with the addition of algae, name and company ,,,								
sushi algae, e.g. nori seaweed, name and company..								
other products with the addition of algae, name and company …….								
**Fish and seafood**								
salmon								
mackerel								

tuna								
cod								
haddock								
plaice								
pollock								
sprat								
herring								
trout								
other fish, name								
Cockles								
Conch								
Crab meat								
Crayfish								
Lobster								
Mussels								
Oysters								
Shrimp								
Other, name								
The most frequently chosen by your types of culinary processing for fish								
cooking in water								
steam cooking (convection oven)								
frying in a pan								
microwave								
baking without foil								
baking in foil								
smoking								
raw, e.g. sushi								
other which …..								
jars								
canned fish								
salted								
other, name								
Meats products								
pork meat								
beef meat								
meat of chicken								
meat of turkey								
meat of duck								
meat of goose								
cold cuts, name ………								
other meat, name……								
The most frequently chosen by your types of culinary processing for fish								
cooking in water								
steam cooking (convection oven)								
frying in a pan								
microwave								
baking without foil								
baking in foil								

smoking								
raw, e.g. Steak tartare								
other which …..								
**Salt**								
Regular table salt, name ….								
Himalayan salt, name…								
Indian salt, name ….								
Sea salt, name …								
other salt, ………..								
**Minerals water and juices**								
iodised water, name ….								
minerals water, name…								
flavored sparkling waters, name….								
fruit juices, name ……..								
vegetable juices, name ……….								
other drinks, name ……								

Supplementary Tables S2. Questions about how salt was added to the food (time of cooking).

	at the end of heating	halfway through heating	at the beginning	I don't salt
groats				
rice				
pasta				
noodles, e.g. gnocchi				
potatoes				
vegetables				
meat				
fish				
meat				
other, name …..				
